# Four-and-a-Half LIM-Domain Protein 2 (FHL2) Induces Neuropeptide Y (NPY) in Macrophages in Visceral Adipose Tissue and Promotes Diet-Induced Obesity

**DOI:** 10.3390/ijms241914943

**Published:** 2023-10-06

**Authors:** Judith Sommer, Hanna Ehnis, Tatjana Seitz, Julia Schneider, Andreas B. Wild, Sandra Moceri, Christa Buechler, Aline Bozec, Georg F. Weber, Susanne Merkel, Ruth Beckervordersandforth, Alexander Steinkasserer, Roland Schüle, Jonel Trebicka, Arndt Hartmann, Anja Bosserhoff, Stephan von Hörsten, Peter Dietrich, Claus Hellerbrand

**Affiliations:** 1 Institute of Biochemistry, Friedrich-Alexander-University Erlangen-Nürnberg, Fahrstr. 17, D-91054 Erlangen, Germany; judith.sommer@fau.de (J.S.); hanna.ehnis@fau.de (H.E.); tatjana.seitz@fau.de (T.S.); julia.schneider@gmx.de (J.S.); ruth.beckervordersandforth@fau.de (R.B.); anja.bosserhoff@fau.de (A.B.); peter.dietrich@fau.de (P.D.); 2 Department of Immune Modulation, University Hospital Erlangen, Hartmannstr. 4, D-91052 Erlangen, Germany; andreas.wild@uk-erlangen.de (A.B.W.); alexander.steinkasserer@uk-erlangen.de (A.S.); 3 Department of Experimental Therapy, Preclinical Experimental Center, University Hospital Erlangen, Friedrich-Alexander-University Erlangen-Nürnberg, Palmsanlage 5, D-91054 Erlangen, Germany; sandramoceri@web.de (S.M.); stephan.v.hoersten@fau.de (S.v.H.); 4 Department of Internal Medicine I, University Hospital of Regensburg, Franz-Josef-Strauß-Allee 11, D-93053 Regensburg, Germany; christa.buechler@klinik.uni-regensburg.de; 5 Department of Internal Medicine 3, Rheumatology and Immunology, University Hospital Erlangen, Friedrich-Alexander-University Erlangen-Nürnberg, Glückstr. 6, D-91054 Erlangen, Germany; aline.bozec@uk-erlangen.de; 6 Department of Surgery, University Hospital Erlangen, Friedrich-Alexander-University Erlangen-Nürnberg, Krankenhausstr. 12, D-91054 Erlangen, Germany; georg.weber@uk-erlangen.de (G.F.W.);; 7 Center for Clinical Research, University of Freiburg Medical School, Breisacherstr. 66, D-79106 Freiburg, Germany; roland.schuele@uniklinik-freiburg.de; 8 Department of Internal Medicine B, University of Münster, Albert-Schweitzer-Campus 1, D-48149 Münster, Germany; jonel.trebicka@ukmuenster.de; 9 Institute of Pathology, Friedrich-Alexander-University Erlangen-Nürnberg, Krankenhausstr. 8/10, D-91054 Erlangen, Germany; arndt.hartmann@uk-erlangen.de

**Keywords:** FHL2, NPY, macrophages, adipocytes, MCP-1, thermogenesis, obesity

## Abstract

Obesity is characterized by the expansion of the adipose tissue, usually accompanied by inflammation, with a prominent role of macrophages infiltrating the visceral adipose tissue (VAT). This chronic inflammation is a major driver of obesity-associated comorbidities. Four-and-a-half LIM-domain protein 2 (FHL2) is a multifunctional adaptor protein that is involved in the regulation of various biological functions and the maintenance of the homeostasis of different tissues. In this study, we aimed to gain new insights into the expression and functional role of FHL2 in VAT in diet-induced obesity. We found enhanced FHL2 expression in the VAT of mice with Western-type diet (WTD)-induced obesity and obese humans and identified macrophages as the cellular source of enhanced FHL2 expression in VAT. In mice with FHL2 deficiency (FHL2^KO^), WTD feeding resulted in reduced body weight gain paralleled by enhanced energy expenditure and uncoupling protein 1 (UCP1) expression, indicative of activated thermogenesis. In human VAT, FHL2 was inversely correlated with UCP1 expression. Furthermore, macrophage infiltration and the expression of the chemokine MCP-1, a known promotor of macrophage accumulation, was significantly reduced in WTD-fed FHL2^KO^ mice compared with wild-type (wt) littermates. While FHL2 depletion did not affect the differentiation or lipid metabolism of adipocytes in vitro, FHL2 depletion in macrophages resulted in reduced expressions of MCP-1 and the neuropeptide Y (NPY). Furthermore, WTD-fed FHL2^KO^ mice showed reduced NPY expression in VAT compared with wt littermates, and NPY expression was enhanced in VAT resident macrophages of obese individuals. Stimulation with recombinant NPY induced not only UCP1 expression and lipid accumulation but also MCP-1 expression in adipocytes. Collectively, these findings indicate that FHL2 is a positive regulator of NPY and MCP-1 expression in macrophages and herewith closely linked to the mechanism of obesity-associated lipid accumulation and inflammation in VAT. Thus, FHL2 appears as a potential novel target to interfere with the macrophage–adipocyte crosstalk in VAT for treating obesity and related metabolic disorders.

## 1. Introduction

Lipids stored in white adipose tissue are the major energy source in mammals. However, an imbalance between energy storage and combustion can lead to excessive fat accumulation, an increase in adipose tissue mass, and obesity [1]. Thus, obesity is a growing epidemic mainly promoted by the Western lifestyle, in particular, overnutrition often aggravated by sparse physical activity [1,2].

Obesity is frequently accompanied by the inflammation of fat tissue with an important role in visceral adipose tissue (VAT). It is caused by adipocyte hypertrophy and hyperplasia with increased infiltration of VAT by macrophages and the elevated production of inflammatory cytokines [3]. This chronic inflammation is a major driver of obesity-associated comorbidities such as insulin resistance and type 2 diabetes, metabolic-associated fatty liver disease, hypertension, and cancer [4]. Thus, the crosstalk between adipocytes and macrophages within white adipose tissue has a significant impact on obesity and related metabolic disorders.

A molecule that plays a pivotal role in the whole body’s energy metabolism is neuropeptide Y (NPY). NPY derived from the brain and sympathetic nerves is a central factor in the regulation of orexigenic activity and energy homeostasis. The exogenous administration or overexpression of NPY stimulates food intake, increases body weight, and promotes adiposity [5,6,7]. More recently, NPY has also been shown to be expressed in adipose tissue and to impact adipocyte metabolism via the control of lipogenesis and lipolysis [8,9]. However, the potential sources and molecular mechanisms through which NPY is associated with adipocyte metabolism are widely unknown.

Four-and-a-half LIM-domain protein 2 (FHL2) is a member of the LIM-only family. Originally, FHL2 has been described as “downregulated in rhabdomyosarcoma LIM protein” (DRAL). The LIM domain protein characteristics enable FHL2 to interact with a variety of other proteins. It is a multifunctional adaptor protein that can interact with an array of cell surface receptors, cytosolic adaptors, structural proteins and kinases [10]. FHL2 lacks the ability to bind to DNA and, therefore, does not directly regulate gene expression. However, FHL2 also interacts with transcription factors and thus is able to modulate and fine-tune gene expression [10]. It is involved in numerous biological activities, including epithelial–mesenchymal transition, cell proliferation, apoptosis, adhesion, migration, and structural stability [11]. A most recent study indicated that FHL2 also affects fat metabolism because FHL2 deficiency in mice reduced high-fat diet-induced obesity [12], but the underlying molecular mechanisms remained unsolved.

In this study, we newly found enhanced FHL2 expression in macrophages in visceral adipose tissue (VAT) of mice with Western-type diet-induced obesity and obese humans. Furthermore, we found that FHL2 induces NPY expression in macrophages and thereby affects lipid metabolism in adipocytes. Moreover, NPY induces the expression of monocyte chemoattractant protein-1 (MCP-1) in adipocytes, and FHL2 also directly enhances MCP-1 expression in macrophages. The chemokine MCP-1 is a strong promotor of macrophage accumulation in obese adipose tissue [13]. These findings suggest that FHL2 in macrophages is a critical regulator of adipocyte–macrophage crosstalk and may be a novel therapeutic target for the treatment of obesity and related metabolic disorders.

## 2. Results

### 2.1. Effect of FHL2 Deficiency on Diet-Induced Obesity

**Figure 1:**
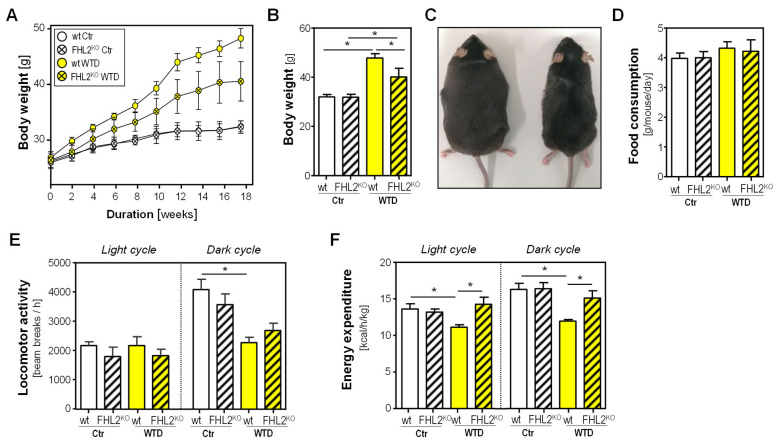
Effect of FHL2 deficiency on diet-induced obesity and energy expenditure. Wt and FHL2^KO^ mice were fed with a Western-type diet (WTD) or standard chow (Ctr) for 18 weeks: (**A**) body weight in the course of the experiment; (**B**) body weight at the end of the experiment; (**C**) representative images of wt (**left**) and FHL2^KO^ (**right**) mice after 18 weeks of WTD feeding; (**D**) food consumption, (**E**) locomotor activity, and (**F**) energy expenditure (EE) analyzed in the PhenoMaster system (* *p* < 0.05).

In order to elucidate the role of FHL2 in obesity, wild-type (wt) and FHL2-deficient (FHL2^KO^) mice were fed with a Western-type diet (WTD) for 18 weeks. As expected, body weight was significantly higher in WTD-fed mice than in control mice receiving standard chow (Ctr) (Figure 1A,B). However, FHL2^KO^ mice gained less body weight, and at the end of the 18-week feeding period, the body weight was significantly lower in FHL2^KO^ than in wt mice fed with WTD (Figure 1A–C). In line with the reduced body weight gain, fasting blood glucose levels were not enhanced in WTD-fed FHL2^KO^ mice but only in WTD-fed Ctr mice (Figure S1A).

The reduced body weight gain of WTD-fed FHL2^KO^ mice could not be explained by different ingestion behavior, since the food consumption of both strains was comparable (Figure 1D).

Furthermore, and as expected, lipid content in the feces of WTD-fed animals was higher than in Ctr mice (Figure S1B). However, there were no differences between genotypes, indicating that intestinal lipid uptake did not differ between FHL2^KO^ and wt mice.

Also, differences in locomotor activity could not explain the WTD-induced weight difference between FHL2^KO^ and wt mice. While locomotor activity was not different during the light cycle, it was reduced in WTD compared with control-fed mice in the dark cycle, but without significant differences between genotypes (Figure 1E). In WTD-fed wt mice, energy expenditure (EE) was significantly decreased compared with wt Ctr mice during light and dark cycles (Figure 1F). However, WTD-fed FHL2^KO^ mice revealed significantly increased EE compared with WTD-fed wt mice during both light and dark cycles (Figure 1F).

In line with the higher EE, the temperature in the cages of FHL2^KO^ mice was significantly higher than in the boxes of the wt mice (Figure S1C). Interestingly, these temperature differences were present in WTD-fed as well as Ctr mice. Overall, the lower box temperature in WTD-fed compared with wt mice can be explained by the lower locomotor activity.

### 2.2. Effect of FHL2 Deficiency on Diet-Induced Expansion and Metabolic Activity of Visceral Adipose Tissue

**Figure 2:**
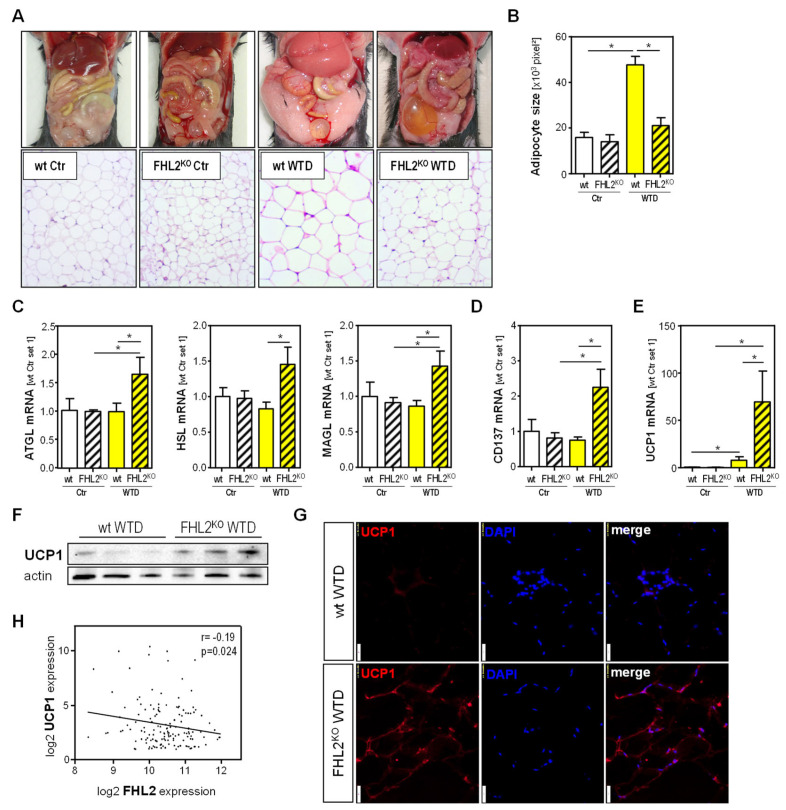
Effect of FHL2 deficiency on diet-induced expansion and metabolic activity of visceral adipose tissue. Wt and FHL2^KO^ mice were fed with a Western-type diet (WTD) or standard chow (Ctr) for 18 weeks: (**A**) macroscopic images (**upper panel**) and HE staining (**lower panel**; 10× magnification) of visceral adipose tissue (VAT); (**B**) adipocyte size; (**C**) ATGL, HSL, and MAGL, (**D**) CD137, and (**E**) UCP1 mRNA expression; (**F**) UCP1 protein level and (**G**) UCP1 immunofluorescence staining (red color) of VAT of wt and FHL2^KO^ WTD mice. DAPI staining (blue) was applied to mark nuclei; (**H**) in silico correlation analysis of FHL2 and UCP1 expression in human VAT (* *p* < 0.05).

Next, we sought to analyze whether metabolic alterations in visceral adipose tissue (VAT) may account for the enhanced EE in FHL2^KO^ mice fed with WTD. The anabolic activity of VAT in response to overnutrition involves adipocyte enlargement and hyperplasia [14]. In line with this, wt mice revealed an apparent VAT expansion (Figure 2A) and significantly increased adipocyte size (Figure 2A,B) in response to WTD feeding. In contrast, WTD-induced VAT expansion was less prominent in FHL2^KO^ mice, and the adipocyte size did not significantly differ between WTD-fed and Ctr FHL2^KO^ mice (Figure 2A,B). Smaller-sized adipocytes are “metabolically more healthy” and have improved metabolic activity [15,16]. In agreement with this, the expression of the lipases adipose triglyceride lipase (ATGL), hormone-sensitive lipase (HSL), and monoacylglycerol lipase (MAGL), which are involved in the intracellular mobilization of fatty acids from triglycerides, was significantly increased in WTD-fed FHL2^KO^ mice (Figure 2C).

The enhanced mobilization of fatty acids and decreased adipocyte size are features of nonshivering thermogenesis [17,18]. CD137 is a specific surface marker for the activation of thermogenesis in white adipocytes (also named beiging). CD137 expression was significantly increased in VAT of FHL2^KO^ compared with wt mice after WTD feeding, indicating increased numbers of beige adipocytes in these animals (Figure 2D). Uncoupling protein 1 (UCP1) is the best-characterized thermogenic effector in VAT, and its expression was significantly induced in response to WTD feeding in wt and FHL2^KO^ mice (Figure 2E). However, this induction was significantly higher in FHL2-deficient animals (Figure 2E–G).

Furthermore, the in silico analysis of human visceral adipose tissue applying the Xena platform [19] revealed a negative correlation between FHL2 and UCP1 expression (Figure 2H). Together, these results indicate an association between FHL2 and reduced UCP1-dependent thermogenesis in VAT and suggest that this association contributes to enhanced energy expenditure and lower fat accumulation in FHL2^KO^ mice in response to WTD feeding.

### 2.3. Effect of FHL2 Depletion in Adipocytes

Our next goal was to analyze whether the observed differences between wt and FHL2^KO^ mice regarding metabolic activity in VAT are caused by the lack of FHL2 in adipocytes. To address this question, we applied the in vitro differentiation model of 3T3-L1 cells.

**Figure 3:**
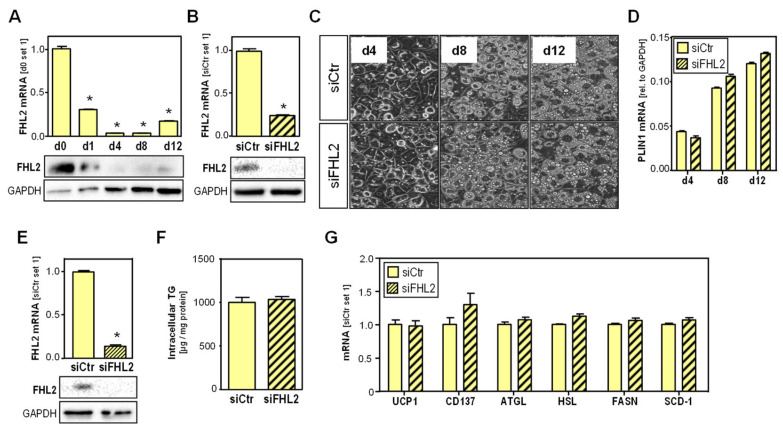
Effect of FHL2 depletion in adipocytes: (**A**) FHL2 mRNA and protein expression in 3T3-L1 cells during differentiation; 3T3-L1 cells were transfected with siPOOLs against FHL2 (siFHL2) or control pools (siCtr) for 72 h at different time points of differentiation; (**B**) FHL2 expression in undifferentiated 3T3-L1 cells; (**C**) light microscopic images during differentiation of 3T3-L1 cells (20x magnification). It can be seen that 3T3-L1 cells with and without FHL2 depletion show a similar differentiation over time; (**D**) PLIN1 mRNA level of FHL2-depleted 3T3-L1 cells during differentiation; (**E**) FHL2 mRNA and protein expression; (**F**) intracellular triglycerides (TG) level; (**G**) UCP1, CD137, ATGL, HSL, FASN, and SCD-1 mRNA expression in fully differentiated 3T3-L1 cells (d14) (* *p* < 0.05).

Interestingly, FHL2 expression rapidly decreased during the in vitro differentiation of 3T3-L1 cells and remained at a low level until d8 (Figure 3A). Furthermore, the RNAi-mediated knockdown of FHL2 (Figure 3B) did not alter the morphology (Figure 3C) or the expression of the adipogenic maker perilipin-1 (PLIN1) (Figure 3D) during the in vitro differentiation of 3T3-L1 cells. These findings suggest that it is unlikely that the reduced expansion of VAT in FHL2^KO^ mice is caused by the impaired differentiation of preadipocytes to adipocytes.

Next, we analyzed the effect of FHL2 knockdown on fully differentiated adipocytes (Figure 3E). Here, FHL2 depletion did not significantly alter intracellular triglyceride levels (Figure 3F). Furthermore, several key markers and enzymes involved in lipid metabolism, including UCP1 and CD137, were not affected by FHL2 depletion (Figure 3G).

Together, these in vitro data suggest that the lack of FHL2 expression in adipocytes is unlikely to be responsible for the observed differences in the metabolic activity in the VAT between wt and FHL2^KO^ mice.

### 2.4. FHL2 Expression in Visceral Adipose Tissue

**Figure 4:**
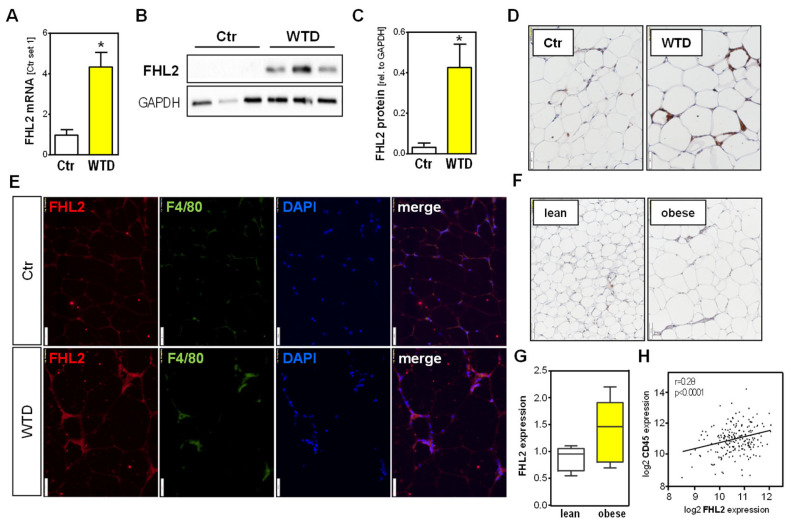
FHL2 expression in visceral adipose tissue. Wild-type mice were fed with a Western-type diet (WTD) or standard chow (Ctr) for 18 weeks. Analysis of (**A**) FHL2 mRNA and (**B**,**C**) FHL2 protein expression in visceral adipose tissue (VAT); (**D**) immunohistochemical staining of FHL2 in VAT tissue (10× magnification); (**E**) co-immunofluorescence staining of FHL2 (red) and F4/80 (green) in VAT (20× magnification); (**F**) immunohistochemical staining of FHL2 in VAT of lean and obese humans (10× magnification); (**G**) in silico analysis of FHL2 expression in VAT of lean and obese children; (**H**) in silico correlation analysis of FHL2 and CD45 expression in human VAT (* *p* < 0.05).

In the next step, we analyzed the FHL2 expression levels in VAT from the mesentery and omentum of mice and found a significant increase in FHL2 in WTD-fed compared with Ctr animals (Figure 4A–C). Immunohistochemical (IHC) FHL2 staining showed pronounced signals in crown-like structures (Figure 4D), which are characteristically formed by macrophages surrounding damaged or necrotic adipocytes [20]. Co-immunofluorescence (Co-IF) staining with the established macrophage marker F4/80 confirmed macrophages as cellular sources of FHL2 in the VAT of WTD-fed mice (Figure 4E). Additionally, in human VAT, FHL2-IHC showed a predominant staining signal in crown-like structures of obese compared with lean individuals (Figure 4F), and FHL2-F4/80 Co-IF confirmed macrophages as cellular sources of FHL2 in human VAT (Figure S2). Furthermore, the in silico analysis of the publicly available dataset GSE9624 through the GEO2R interface (http://www.ncbi.nlm.nih.gov/geo/geo2r/, accessed on 4 April 2023) revealed a trend for higher FHL2 expression in the VAT of obese children than in that of lean children (Figure 4G). Moreover, by applying the Xena platform, we found a significant correlation between the expression of FHL2 and the macrophage marker CD45 in adult human VAT [19] (Figure 4H).

Together, these results reveal the upregulation of FHL2 in the VAT of mice with WTD-induced obesity, as well as in the VAT of obese men, and indicate macrophages as the main cellular source of enhanced FHL2 expression.

### 2.5. Effect of FHL2 Depletion in Macrophages

**Figure 5:**
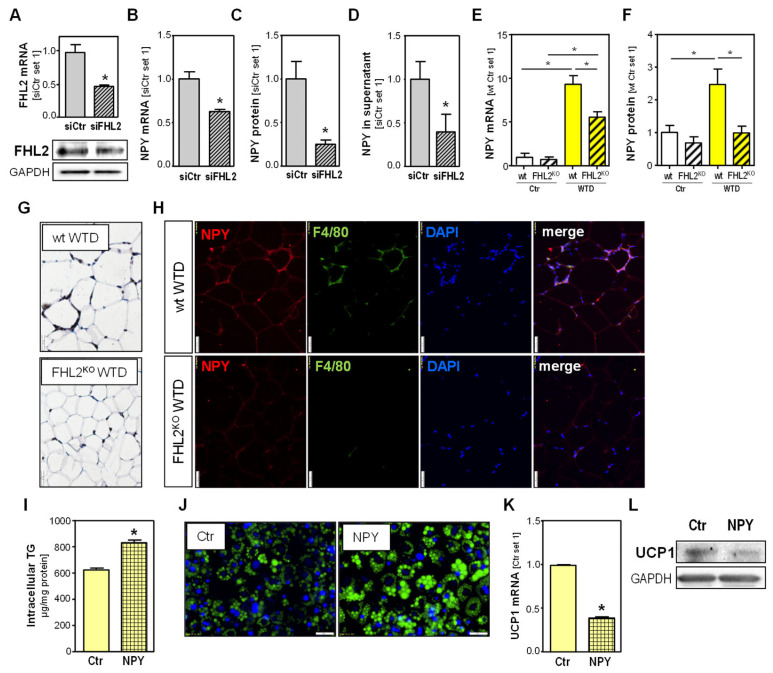
Effect of FHL2 depletion on NPY expression in macrophages and visceral adipose tissue and effect of NPY on adipocytes: (**A**) FHL2 mRNA and protein expression. (**B**) NPY mRNA expression, (**C**) NPY protein levels in cell lysates, and (**D**) NPY protein levels in supernatants of Raw246.7 cells with RNAi-mediated FHL2 depletion (siFHL2) and control cells (siCtr); (**E**) NPY mRNA and (**F**) NPY protein level; (**G**) immunohistochemical NPY staining (10× magnification); (**H**) co-immunofluorescence staining of NPY (red) and F4/80 (green) (20× magnification) in visceral adipose tissue of wild-type (wt) and FHL2^KO^ mice fed with a Western-type diet (WTD) or standard chow (Ctr) for 18 weeks; (**I**) intracellular triglycerides, (**J**) BODIPY staining (20× magnification), (**K**) UCP1 mRNA, and (**L**) UCP-1 protein levels in differentiated 3T3-L1 cells treated with NPY (100 ng/mL) for 24 h (* *p* < 0.05).

Next, we aimed to search for the potential mechanism of how the lack of FHL2 in macrophages in VAT may account for the observed phenotype of FHL2^KO^ mice. The neuropeptide Y (NPY) has been found to have anti-lipolytic activity and to impair thermogenesis [21]. Furthermore, recent studies revealed that NPY is also locally expressed in VAT [22]. Interestingly, RNAi-mediated FHL2 depletion in the murine macrophage cell line RAW 246.7 (Figure 5A) resulted in the significant downregulation of NPY expression and secretion of NPY (Figure 5B–D). The knockdown of FHL2 in primary murine peritoneal macrophages confirmed a significantly decreased NPY expression (Figure S3A–D).

The VAT analysis of FHL2^KO^ and wt mice showed increased NPY expression in both genotypes after WTD feeding compared with standard chow, but this NPY induction was significantly lower in FHL2-deficient than in wt animals (Figure 5E,F). IHC analysis showed NPY staining in crown-like structures in the VAT of WTD-fed animals, with a weaker staining signal in FHL2^KO^ than in wt mice (Figure 5G), and the Co-IF staining of NPY and F4/80 revealed macrophages as cellular sources of NPY (Figure 5H). The Co-IF staining of human VAT revealed macrophages as the main cellular source of NPY in obese individuals, while only a very weak immunosignal was detectable in the VAT of lean persons (Figure S3E).

To gain insight into the effect of NPY on adipocyte metabolism, we treated fully differentiated 3T3-L1 adipocytes with recombinant NPY. NPY stimulation significantly increased the intracellular triglyceride levels (Figure 5I), and BODIPY staining also showed enhanced lipid accumulation in NPY-stimulated cells (Figure 5J). Moreover, and in agreement with the in vivo data in the VAT of mice, we observed the downregulation of UCP1 expression in NPY-treated cells (Figure 5K,L).

Together, these findings suggest that FHL2-induced NPY expression in macrophages affects the lipid metabolism in adipocytes, at least in part by inhibiting the thermogenic lipolysis machinery.

### 2.6. Effect of FHL2 Deficiency on Diet-Induced Inflammation of Visceral Adipose Tissue

**Figure 6:**
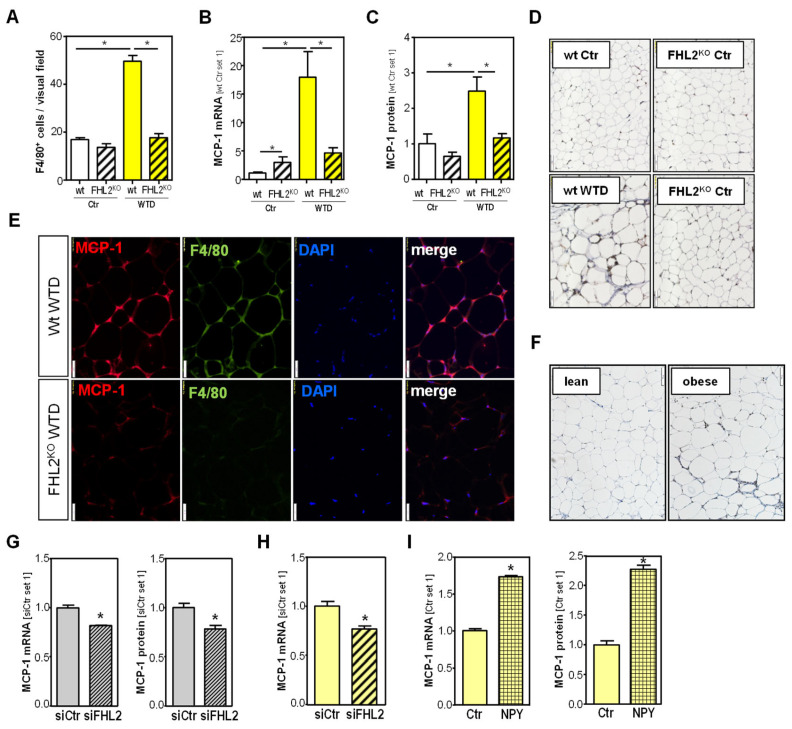
Effect of FHL2 depletion on MCP-1 expression in visceral adipose tissue (VAT), macrophages, and adipocytes: (**A**) F4/80-positive cells; (**B**) MCP-1 mRNA expression, (**C**) MCP-1 protein levels, (**D**) immunohistochemical MCP-1 staining (10× magnification), and (**E**) co-immunofluorescence staining of MCP-1 (red) and F4/80 (green) (20× magnification) in VAT of wild-type (wt) and FHL2^KO^ mice fed with a Western-type diet (WTD) or standard chow (Ctr) for 18 weeks. DAPI staining (blue) was used to visualize cell nuclei. In VAT of wt mice, one can recognize the co-localization of MCP-1 and F4/80 indicating macrophages as cellular sources of MCP-1 in VAT. In FHL2-ko mice, both MCP-1 and F4/80 were significantly lower than in VAT of wt mice; (**F**) immunohistochemical MCP-1 staining in VAT of obese and lean humans (10× magnification); (**G**) MCP-1 mRNA and protein level in Raw246.7 cells transfected with siPOOLs against FHL2 (siFHL2) or control pools (siCtr) for 72 h; (**H**) MCP-1 mRNA level in differentiated 3T3-L1 cells transfected with siPOOLs against FHL2 (siFHL2) or control pools (siCtr) for 72 h; (**I**) MCP-1 mRNA and MCP-1 protein levels in 3T3-L1 cells treated with NPY (100 nM) for 24 h (*: *p* < 0.05).

Besides the apparent expansion of the adipose tissue, obesity is known to lead to increased macrophage infiltration into the VAT, and our IF analysis also showed more macrophages in the VAT of WTD-fed animals than in the VAT of control-fed mice (Figure 4E and Figure 5H), as well as in the VAT of obese compared with lean humans (Figures S2 and S3E). However, we noticed significantly fewer F4/80-positive cells in the VAT of WTD-fed FHL2^KO^ mice than in the VAT of WTD-fed wt mice (Figure 6A), as well as in obese compared with lean human individuals (Figure S4A). The chemokine MCP-1 is known to play an important role in macrophage attraction to the VAT [23], and in the VAT of WTD-fed mice, we also found significantly increased MCP-1 expression (Figure 6B,C). However, interestingly, the WTD-induced MCP-1 expression was significantly lower in FHL2^KO^ mice (Figure 6B,C). MCP-1 IHC confirmed a stronger staining signal in the wt mice than in FHL2^KO^ mice after WTD feeding (Figure 6D). The MCP-1 staining signal was mainly localized in crown-like structures, and Co-IF for MCP-1 and F4/80 revealed macrophages as the main cellular source of MCP-1 in the VAT of WTD-fed mice (Figure 6E). In human VAT, MCP-1 IH analysis revealed a stronger staining signal in tissues from obese compared with lean individuals, and here as well, staining was mainly localized in crown-like structures (Figure 6F).

To validate whether the lack of FHL2 is responsible for reduced MCP-1 expression in the macrophages in the VAT of FHL2^KO^ mice, we analyzed RAW246.7 cells after FHL2 depletion. Here, we found significantly reduced MCP-1 expression and secretion in FHL2-depleted cells (Figure 6G and Figure S4B). Reduced MCP-1 expression was confirmed in FHL2-depleted primary murine macrophages (Figure S4C,D). Although IHC and IF revealed macrophages as the main cellular source of MCP-1 in the VAT, we wanted to analyze whether FHL2 deficiency may additionally impact MCP-1 expression in adipocytes, because previous studies have reported that adipocytes also express this chemokine [24,25]. The analysis of FHL2-depleted and differentiated 3T3-L1 cells revealed reduced MCP-1 expression compared with control cells (Figure 6H). Moreover, we found that NPY stimulation significantly induced MCP-1 expression and secretion in differentiated 3T3-L1 cells (Figure 6I and Figure S4E). Together, these data indicate that FHL2 induces MCP-1 in VAT directly via transcriptional regulation in macrophages and adipocytes. Furthermore, the macrophages of wt mice express higher levels of NPY than those of FHL2-deficient mice, leading to an increased MCP-1 expression in adipocytes, which in turn results in the recruitment of more NPY-expressing macrophages to the VAT, leading to a vicious circle.

## 3. Discussion

FHL2 interacts with a variety of other proteins. Herewith, it is involved in the regulation of various biological functions and in processes through which homeostasis is maintained in different tissues [11]. Conversely, the dysregulation of FHL2 has been shown in different pathological conditions, including wound healing, inflammation, and cancer [11]. In this study, we aimed to gain new insights into the expression and the functional role of FHL2 in adipose tissue and obesity.

We found enhanced FHL2 expression in visceral adipose tissue (VAT) of mice with Western-type diet (WTD)-induced obesity and obese humans. In mice with FHL2 deficiency, WTD feeding resulted in reduced body weight gain without food intake alterations. As a potential mechanism, we found enhanced energy expenditure and higher UCP1 expression in VAT. The mitochondrial protein UCP1 is a regulator of nonshivering thermogenesis. It uncouples the respiration of cells and their mitochondrial ATP synthesis to dissipate energy in the form of heat. The activation of thermogenesis in adipose tissue is inversely correlated with body weight and adiposity [26], and thus, its enhanced expression appears as a potential explanation for the reduced weight gain in FHL2^KO^ mice. However, FHL2 depletion did not affect differentiation, lipid accumulation, or UCP1 expression in adipocytes. Thus, in search for the molecular mechanisms through which FHL2 deficiency protects from diet-induced obesity, we focused our attention on macrophages, which we identified as the main cellular source of the enhanced FHL2 expression in the VAT of obese mice and humans. We found that FHL2 depletion reduced NPY expression in macrophages, and in line with this, NPY expression was significantly reduced in the VAT of FHL2^KO^ compared with WTD pair-fed control mice.

Most previous studies analyzing NPY effects on lipid metabolism in adipose tissue have focused on NPY derived from the bloodstream/brain or sympathetic nerve terminals that innervate the adipose tissue [5,27]. Our study indicates that local-macrophage-derived NPY may also impact the pathogenesis of obesity.

One major limitation of our study is the use of mice with systemic FHL2^KO^. Thus, it has to be considered that, besides VAT, the lack of FHL2 in other organs and fat compartments also contributes to the observed phenotype. Indeed, a recent study by Clemente-Olivo et al. provided the first hint that FHL2 affects browning in subcutaneous and perigonadal white adipose tissue [12]. However, these authors did not observe signs of browning in VAT [12]. Potential reasons for this discrepancy with our study may be the significantly shorter feeding period of 8 weeks (vs. 18 weeks in this study) and the application of a high-fat diet for the induction of obesity. Here, we applied a WTD that, in addition to a higher content of fatty acids, also contained elevated amounts of cholesterol and fructose to more closely mimic the typical type of overnutrition in most Western countries [28]. Thus, one may speculate that the impact of FHL2 in different fat storage compartments may vary during the course of diet-induced weight gain and associated pathological changes. Moreover, Clemente-Olivo et al. found an increased cardiac glucose uptake in FHL2^KO^ mice as a further potential explanation of enhanced energy expenditure and the reduced expansion of adipose tissue upon feeding an obesity-inducing high-fat diet [12]. Studies with cell-type specific FHL2 depletion are required to unravel the complex role of FHL2 in different tissues as well as their quantitative effects on the development of obesity and its comorbidities.

Besides the obvious enlargement of fat depots, a chronic inflammatory state of the adipose tissue is another key feature of obesity. Inflammation occurs in the expanding adipose tissue and is coupled with the infiltration of immune cells. Macrophages remain the prominent immune cell type in inflamed obese VAT, largely due to the recruitment of monocytes from the circulation [29], and the chemokine MCP-1 is a strong promotor of macrophage accumulation in the obese adipose tissue [13,23].

Several studies have shown an influence of FHL2 on the immune cell infiltration of injured tissues [11]. Still, the exact role of FHL2 in these processes is somewhat controversial [11]. Obesity-induced metabolic changes in VAT can be considered as tissue injury, and here, we found significantly reduced MCP-1 expression in the VAT of FHL2^KO^ mice compared with wt controls after feeding an obesity-inducing WTD. Firstly, we found reduced MCP-1 expression in FHL2-depleted macrophages, suggesting that FHL2 induces MCP-1 expression in macrophages and, herewith, promotes a vicious circle of inflammation in VAT. Moreover, we found that NPY induced MCP-1 expression in adipocytes in vitro. Even if the MCP-1 expression levels in adipocytes in VAT appear lower than the level in macrophages, indirect NPY-mediated FHL2 effects on adipocytes may additionally contribute to the recruitment of monocytes and, herewith, the perpetuation of a chronic inflammatory state in the VAT.

**Figure 7:**
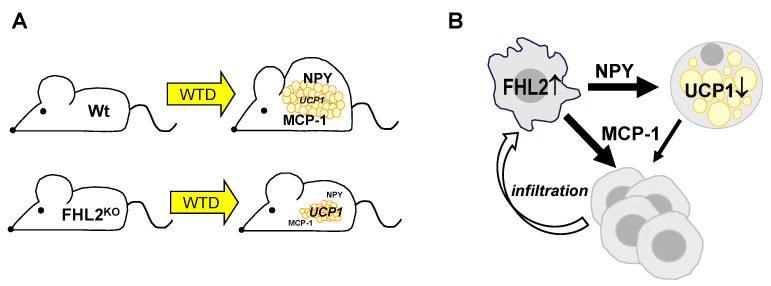
Impact of FHL2 deficiency on diet-induced obesity and proposed mechanisms of the role of FHL2 in the crosstalk between macrophages and adipocytes in visceral adipose tissue: (**A**) mice with FHL2 deletion (FHL2^KO^) are protected against Western-type diet (WTD)-induced weight gain and show reduced expression of NPY and MCP-1 in visceral adipose tissue (VAT) compared with pair-fed wild-type (Wt) mice. Furthermore, FHL2^KO^ mice show enhanced expression of the nonshivering thermogenic regulator, UCP1, and in agreement with this, enhanced energy expenditure; (**B**) macrophages are the cellular source of enhanced FHL2 expression in VAT, and the depletion of FHL2 in macrophages reduces their NPY as well as MCP-1 expression. In adipocytes, NPY inhibits UCP1 expression but induces MCP-1 expression and secretion. Both, MCP-1 from macrophages and adipocytes, may contribute to further recruitment of monocytes to the VAT, perpetuating and enhancing a vicious cycle.

In conclusion, our results highlight the beneficial effects of FHL2 antagonism on adiposity and adiposity-associated inflammation. Future studies with cell-specific knockout of FHL2 are necessary to unwire the detailed role of FHL2 in different cells and tissues. Still, in combination with our supporting in vitro data, we would like to suggest a critical role of FHL2 in the crosstalk between macrophages and adipocytes in the VAT during diet-induced obesity (Figure 7). Collectively, our findings indicate that FHL2, as a positive regulator of NPY and MCP-1, is closely linked to the mechanism of obesity-associated lipid accumulation and inflammation in VAT. These findings suggest that FHL2 in macrophages may be a novel therapeutic target not only to reduce obesity but also to prevent highly prevalent complications.

## 4. Materials and Methods

### 4.1. Cells and Cell Culture

For this study, 3T3-L1 preadipocytes (Kerafast, Boston, MA, USA) were provided by the Bozec laboratory (Friedrich-Alexander-Universität Erlangen–Nürnberg and University Hospital Erlangen, Erlangen, Germany). Cell culture and differentiation procedure of 3T3-L1 preadipocytes was performed as described [30,31]. Briefly, cells were grown to 80% confluence and differentiated into adipocytes in 10% DMEM supplemented with 0.5 mM 3-isobutyl-1-methylxanthin (IBMX), 1 µg/mL insulin, and 0.25 µM dexamethasone for 4 days. From day 5 onwards, cells were cultured in 10% DMEM supplemented with 1µg/mL insulin (adipocyte maintenance medium, AMM). The differentiated phenotype was demonstrated by the typical intracellular accumulation of lipid droplets, as controlled via light microscopy.

Murine RAW 246.7 macrophages (ATCC, Manassas, VA, USA) were provided by the Steinkasserer laboratory (University Hospital Erlangen, Erlangen, Germany). Cells were cultured at 37 °C and 8% CO_2_ in DMEM (Sigma, Deisenhofen, Germany), supplemented with 10% fetal bovine serum (PAN, Aidenbach, Germany) and 1% penicillin–streptomycin (Sigma, Deisenhofen, Germany) (10% DMEM).

The isolation of primary murine peritoneal macrophages was performed by carefully removing the abdominal skin and injecting 5 mL of cold PBS with 5 mM EDTA into the peritoneal cavity. Subsequently, the fluid was aspirated, and the obtained peritoneal macrophages were used for experiments. Cells were cultured at 37 °C and 8% CO_2_ in 10% DMEM.

Transfection with FHL2 siRNA pools was performed as described in [32] by using the Lipofectamine RNAiMAX transfection reagent (Life Technologies, Darmstadt, Germany) and siRNA pools against FHL2 mRNA (siTOOLs Biotech GmbH, Planegg, Germany). For stimulation experiments, cells were transferred to serum-free AMM and treated with recombinant NPY (Merck, Darmstadt, Germany) at a concentration of 100 nM [8].

### 4.2. Animal Experiments

Male FHL2 knockout (Fhl2^KO^) mice [33] and wild-type (wt) littermates (n = 7–8/group) were fed with a Western-type diet (WTD) [34] containing 38% fat, 30% sucrose, and 0.2% cholesterol (Ssniff, Soest, Germany) starting at the age of 8 weeks. Control (Ctr) mice were fed with standard chow, and all mice had access to water ad libitum. After 18 weeks, animals were sacrificed, and blood samples were collected. Tissue samples were either fixed in 5% formalin or snap-frozen in liquid nitrogen and stored at −80 °C until subsequent analyses. The animal studies were approved by the Committee for Animal Health and Care of the local government (55.2-2532-2-1374) and conformed to international guidelines on the ethical use of animals.

The PhenoMaster system (TSE Systems GmbH, Bad Homburg, Germany) was used for metabolic screening in a home-cage-like environment, as previously described [35]. Briefly, mice were singly housed in an experimental cage for a period of 72 h, and data were continuously collected from single animals. Experiments were performed between 14 and 16 weeks of feeding and under standard housing and light-cycle conditions. Food consumption and water consumption were measured, and spontaneous locomotion and rearing events were detected using infrared light beam frames surrounding each experimental cage. Respiratory gas analysis was achieved via an O_2_/CO_2_ gas sensor pair that allowed for the automated calculation of O_2_ consumption, CO_2_ production, and energy expenditure (kcal/h/kg) for each animal (performed using TSE PhenoMaster software, version 4.4.6). The gas sensor pair was calibrated with calibration gas mixtures before each test session. After the experiment, all animals were reunified in their original social groups. Only data collected between the first and third dark phases were pooled since habituation processes might bias data derived from the first (incomplete) light phase/first 8 h of measurement.

Blood glucose concentrations were measured in samples taken from the tail vein using an Accutrend glucometer (Roche, Mannheim, Germany).

### 4.3. Human Visceral Adipose Tissue

Paraffin-embedded visceral adipose tissue (VAT) samples were obtained during standard operating procedures from patients with sigmoidal colon cancer during tumor resection. Samples were obtained from 10 patients with a body mass index (BMI) of 20.8–24.9 kg/m² (mean: 23.0 kg/m²), defined as normal weight, and 10 patients with BMI 35.1–46.1 kg/m² (mean: 40.3 kg/m²), defined as class II-III obesity (WHO). The study was approved by the Clinical Ethics Committee of the Friedrich-Alexander-Universität (FAU) Erlangen–Nürnberg (132_20 Bc).

### 4.4. Histological, Immunohistochemical, and Immunofluorescence Analysis

For hematoxylin and eosin (HE) staining, immunohistochemical, or immunofluorescence analysis, standard 5 µm sections of formalin-fixed and paraffin-embedded tissue blocks were used. Immunohistochemical staining was performed as described in [34], using the following primary antibodies: rat anti-F4/80 (BLD-123101, 1:200, BioLegend, San Diego, CA, USA); rabbit anti-FHL2 (provided by the Schüle Laboratory, University of Freiburg, Freiburg, Germany); mouse anti-MCP-1 (MA5-17040, 1:100, Invitrogen, Thermo Fisher Scientific, Waltham, MA, USA); sheep anti-NPY (ab6173, 1:200, Abcam, Cambridge, UK); and rabbit anti-UCP1 (#14670, 1:200, Cell Signaling Technology, Danvers, MA, USA). For immunofluorescence, Alexa Fluor 546-conjugated goat anti-rabbit IgG (A11010, 1:1000; Invitrogen, Thermo Fisher Scientific); FITC-conjugated rabbit anti-rat IgG (F1763, 1:500, Sigma); and Cy3- conjugated donkey anti-sheep IgG (713-165-003, 1:1000, Jackson ImmunoResearch Laboratories, Inc., West Grove, PA, USA) were used as secondary antibodies.

Microscopic images were taken, analyzed, and processed using an Olympus™ IX83 microscope with the cellSens Dimension software (Olympus Soft Imaging Solutions GmbH, Münster, Germany). Adipocyte size was analyzed using the open source software ImageJ as described in [36].

### 4.5. Analysis of Lipid Content

Total cellular triglycerides were extracted and quantified with a triglyceride-hit (PAP) kit (BIOMED, Oberschleißheim, Germany) as described in [37]. The BODIPY staining of intracellular lipids was performed as described in [38], with slight modifications. Briefly, cells were fixed with 4% PFA for 10 min and incubated with BODIPY (2 µg/mL) and DAPI (10 µg/mL) in PBS for 45 min at 37 °C. Cells were washed to remove excess staining and subsequently imaged.

### 4.6. RNA Analysis

RNA isolation from cells and tissues and subsequent reverse transcription were performed as described in [32]. Quantitative real-time PCR was performed by applying LightCycler technology (Roche Diagnostics, Mannheim, Germany) while using specific sets of primers, as listed in Table 1. For normalization, the amplification of cDNA derived from β-actin was carried out.

### 4.7. Protein Analysis

Protein extraction and Western blotting were performed as described in [32] by applying the following primary antibodies: mouse anti-actin (MAB1501, 1:10,000, Merck Millipore); rabbit anti-Fhl2 (provided by the Schüle Laboratory, University of Freiburg, Freiburg im Breisgau, Germany); rabbit anti-GAPDH (#2118, 1:1000, Cell Signaling); mouse anti-MCP-1 (MA5-17040, 1:1000, Invitrogen); and rabbit anti-UCP1 (#14640, 1:1000, Cell Signaling). Mouse anti-rabbit (sc-2357, 1:10,000, Santa Cruz Biotechnology, Dallas, TX, USA) and horse anti-mouse (#7076, 1:3000, Cell Signaling) were used as secondary antibodies.

For the quantification of NPY and MCP-1 in tissue and cell protein lysates or supernatants, commercially available mouse NPY (Merck, Darmstadt, Germany) and CCL2/MCP-1 (R&D Systems, Minneapolis, MN, USA) ELISA kits were used according to the manufacturer’s instructions.

### 4.8. Statistical Analysis

Values are presented as mean ± SEM. Comparison between groups was performed using Student’s unpaired *t*-test, or when appropriate, a one-way ANOVA test. A *p* value < 0.05 was considered statistically significant. All in vitro analyses were performed at least in triplicates. Calculations were performed using the statistical computer package GraphPad Prism version 6.01 for Windows (GraphPad Software, San Diego, CA, USA).

## Figures and Tables

**Table 1:** Primer sequences for quantitative real-time PCR.

Gene	Forward (5′-3′)	Reverse (5′-3′)
ATGL	CAACGCCACTCACATCTACGGA	CAGGTTGAAGGAGGGATGCAGA
β-actin	AGGCCAACCGTGAAAAGAT	GGCGTGAGGGAGAGCATA
CD137	CTCGCTGCCCTGAGATCGAA	TCGGCTGTCCACCTATGCTG
FASN	ACAATGGACCCCCAGCTTCG	CAGACGCCAGTGTTCGTTCC
FHL2	ACTGCCTGACCTGCTTCTGT	TTGCCTGGTTATGAAAGAAAA
GAPDH	AGCCTCGTCCCGTAGACAAA	TGATGGGCTTCCCGTTGATG
HSL	TGGCAGTGGTGTGTAACTAGGATT	TCGTGCGTAAATCCATGCTGTG
MAGL	CTGTGGCGGTAGTGGAATGC	CCCAGCTCCATGGGACACAA
MCP-1	TGCAGGTCCCTGTCATGCTTC	TGGACCCATTCCTTCTTGGGGT
NPY	TGGCCAGATACTACTCCGCT	GCAGACTGGTTTCAGGGGAT
PLIN1	CTTGGGCGTCTGCCTTACCTA	TTGCTGGCACCCTGTACACC
SCD-1	AACAGTGCCGCGCATCTCTA	GAAGCCCAAAGCTCAGCTACTC
UCP1	ACACCAAGGAAGGACCGACG	ATGACGTTCCAGGACCCGAG

## Data Availability

All data generated in this study are included in the article and the Supplementary Material Files and will be made available upon reasonable request.

## Supplementary Materials

The following supporting information can be downloaded at: https://www.mdpi.com/article/10.3390/ijms241914943/s1.

## References

[1] Loffler M.C., Betz M.J., Blondin D.P., Augustin R., Sharma A.K., Tseng Y.H., Scheele C., Zimdahl H., Mark M., Hennige A.M. (2021). Challenges in Tackling Energy Expenditure as Obesity Therapy: From Preclinical Models to Clinical Application. Mol. Metab..

[2] Saklayen M.G. (2018). The Global Epidemic of the Metabolic Syndrome. Curr. Hypertens. Rep..

[3] Sun K., Kusminski C.M., Scherer P.E. (2011). Adipose Tissue Remodeling and Obesity. J. Clin. Investig..

[4] Longo M., Zatterale F., Naderi J., Parrillo L., Formisano P., Raciti G.A., Beguinot F., Miele C. (2019). Adipose Tissue Dysfunction as Determinant of Obesity-Associated Metabolic Complications. Int. J. Mol. Sci..

[5] Kuo L.E., Kitlinska J.B., Tilan J.U., Li L., Baker S.B., Johnson M.D., Lee E.W., Burnett M.S., Fricke S.T., Kvetnansky R. (2007). Neuropeptide Y Acts Directly in the Periphery on Fat Tissue and Mediates Stress-Induced Obesity and Metabolic Syndrome. Nat. Med..

[6] Ruohonen S.T., Pesonen U., Moritz N., Kaipio K., Roytta M., Koulu M., Savontaus E. (2008). Transgenic Mice Overexpressing Neuropeptide Y in Noradrenergic Neurons: A Novel Model of Increased Adiposity and Impaired Glucose Tolerance. Diabetes.

[7] Ruohonen S.T., Vähätalo L.H., Savontaus E. (2012). Diet-Induced Obesity in Mice Overexpressing Neuropeptide Y in Noradrenergic Neurons. Int. J. Pept..

[8] Park S., Fujishita C., Komatsu T., Kim S.E., Chiba T., Mori R., Shimokawa I. (2014). Npy Antagonism Reduces Adiposity and Attenuates Age-Related Imbalance of Adipose Tissue Metabolism. FASEB J..

[9] Park S., Komatsu T., Kim S.E., Tanaka K., Hayashi H., Mori R., Shimokawa I. (2017). Neuropeptide Y Resists Excess Loss of Fat by Lipolysis in Calorie-Restricted Mice: A Trait Potential for the Life-Extending Effect of Calorie Restriction. Aging Cell.

[10] Tran M.K., Kurakula K., Koenis D.S., de Vries C.J. (2016). Protein-Protein Interactions of the Lim-Only Protein Fhl2 and Functional Implication of the Interactions Relevant in Cardiovascular Disease. Biochim. Biophys. Acta.

[11] Wixler V. (2019). The Role of Fhl2 in Wound Healing and Inflammation. FASEB J..

[12] Clemente-Olivo M.P., Habibe J.J., Vos M., Ottenhoff R., Jongejan A., Herrema H., Zelcer N., Kooijman S., Rensen P.C., van Raalte D.H. (2021). Four-and-a-Half Lim Domain Protein 2 (Fhl2) Deficiency Protects Mice from Diet-Induced Obesity and High Fhl2 Expression Marks Human Obesity. Metab. Clin. Exp..

[13] Engin A.B. (2017). Adipocyte-Macrophage Cross-Talk in Obesity. Adv. Exp. Med. Biol..

[14] Jo J., Gavrilova O., Pack S., Jou W., Mullen S., Sumner A.E., Cushman S.W., Periwal V. (2009). Hypertrophy and/or Hyperplasia: Dynamics of Adipose Tissue Growth. PLoS Comput. Biol..

[15] Honecker J., Ruschke S., Seeliger C., Laber S., Strobel S., Pröll P., Nellaker C., Lindgren C.M., Kulozik U., Ecker J. (2022). Transcriptome and Fatty-Acid Signatures of Adipocyte Hypertrophy and Its Non-Invasive Mr-Based Characterization in Human Adipose Tissue. EBioMedicine.

[16] Landgraf K., Rockstroh D., Wagner I.V., Weise S., Tauscher R., Schwartze J.T., Löffler D., Bühligen U., Wojan M., Till H. (2015). Evidence of Early Alterations in Adipose Tissue Biology and Function and Its Association with Obesity-Related Inflammation and Insulin Resistance in Children. Diabetes.

[17] Sanchez-Gurmaches J., Hung C.M., Guertin D.A. (2016). Guertin. Emerging Complexities in Adipocyte Origins and Identity. Trends Cell Biol..

[18] Wu L., Zhang L., Li B., Jiang H., Duan Y., Xie Z., Shuai L., Li J., Li J. (2018). Amp-Activated Protein Kinase (Ampk) Regulates Energy Metabolism through Modulating Thermogenesis in Adipose Tissue. Front. Physiol..

[19] Goldman M.J., Craft B., Hastie M., Repečka K., McDade F., Kamath A., Banerjee A., Luo Y., Rogers D., Brooks A.N. (2020). Visualizing and Interpreting Cancer Genomics Data Via the Xena Platform. Nat. Biotechnol..

[20] Cinti S., Mitchell G., Barbatelli G., Murano I., Ceresi E., Faloia E., Wang S., Fortier M., Greenberg A.S., Obin M.S. (2005). Adipocyte Death Defines Macrophage Localization and Function in Adipose Tissue of Obese Mice and Humans. J. Lipid Res..

[21] Zhang W., Cline M.A., Gilbert E.R. (2014). Hypothalamus-Adipose Tissue Crosstalk: Neuropeptide Y and the Regulation of Energy Metabolism. Nutr. Metab..

[22] Yang K., Guan H., Arany E., Hill D.J., Cao X. (2008). Neuropeptide Y Is Produced in Visceral Adipose Tissue and Promotes Proliferation of Adipocyte Precursor Cells Via the Y1 Receptor. FASEB J..

[23] Kanda H., Tateya S., Tamori Y., Kotani K., Hiasa K.I., Kitazawa R., Kitazawa S., Miyachi H., Maeda S., Egashira K. (2006). Mcp-1 Contributes to Macrophage Infiltration into Adipose Tissue, Insulin Resistance, and Hepatic Steatosis in Obesity. J. Clin. Investig..

[24] Gerhardt C.C., Romero I.A., Cancello R., Camoin L., Strosberg A.D. (2001). Chemokines Control Fat Accumulation and Leptin Secretion by Cultured Human Adipocytes. Mol. Cell. Endocrinol..

[25] Sartipy P., Loskutoff D.J. (2003). Monocyte Chemoattractant Protein 1 in Obesity and Insulin Resistance. Proc. Natl. Acad. Sci. USA.

[26] van Marken Lichtenbelt W.D., Vanhommerig J.W., Smulders N.M., Drossaerts J.M., Kemerink G.J., Bouvy N.D., Schrauwen P., Teule G.J. (2009). Cold-Activated Brown Adipose Tissue in Healthy Men. N. Engl. J. Med..

[27] Chao P.T., Yang L., Aja S., Moran T.H., Bi S. (2011). Knockdown of Npy Expression in the Dorsomedial Hypothalamus Promotes Development of Brown Adipocytes and Prevents Diet-Induced Obesity. Cell Metab..

[28] Christ A., Lauterbach M., Latz E. (2019). Western Diet and the Immune System: An Inflammatory Connection. Immunity.

[29] Kolb H. (2022). Obese Visceral Fat Tissue Inflammation: From Protective to Detrimental?. BMC Med..

[30] Gaculenko A., Gregoric G., Popp V., Seyler L., Ringer M., Kachler K., Wu Z., Kisel W., Hofbauer C., Hofbauer L.C. (2021). Systemic Pparγ Antagonism Reduces Metastatic Tumor Progression in Adipocyte-Rich Bone in Excess Weight Male Rodents. J. Bone Miner. Res..

[31] Bauer S., Wanninger J., Schmidhofer S., Weigert J., Neumeier M., Dorn C., Hellerbrand C., Zimara N., Schaffler A., Aslanidis C. (2011). Sterol Regulatory Element-Binding Protein 2 (Srebp2) Activation after Excess Triglyceride Storage Induces Chemerin in Hypertrophic Adipocytes. Endocrinology.

[32] Sommer J., Dorn C., Gäbele E., Bataille F., Freese K., Seitz T., Thasler W.E., Büttner R., Weiskirchen R., Bosserhoff A. (2020). Four-and-a-Half Lim-Domain Protein 2 (Fhl2) Deficiency Aggravates Cholestatic Liver Injury. Cells.

[33] Wixler V., Hirner S., Muller J.M., Gullotti L., Will C., Kirfel J., Gunther T., Schneider H., Bosserhoff A., Schorle H. (2007). Deficiency in the Lim-Only Protein Fhl2 Impairs Skin Wound Healing. J. Cell Biol..

[34] Dorn C., Engelmann J.C., Saugspier M., Koch A., Hartmann A., Müller M., Spang R., Bosserhoff A., Hellerbrand C. (2014). Increased Expression of C-Jun in Nonalcoholic Fatty Liver Disease. Lab. Investig..

[35] Urbach Y.K., Raber K.A., Canneva F., Plank A.C., Andreasson T., Ponten H., Kullingsjo J., Nguyen H.P., Riess O., von Horsten S. (2014). Automated Phenotyping and Advanced Data Mining Exemplified in Rats Transgenic for Huntington’s Disease. J. Neurosci. Methods.

[36] Parlee S.D., Lentz S.I., Mori H., MacDougald O.A. (2014). Quantifying Size and Number of Adipocytes in Adipose Tissue. Methods Enzymol..

[37] Wobser H., Dorn C., Weiss T.S., Amann T., Bollheimer C., Büttner R., Schölmerich J., Hellerbrand C. (2009). Lipid Accumulation in Hepatocytes Induces Fibrogenic Activation of Hepatic Stellate Cells. Cell Res..

[38] Targett-Adams P., Chambers D., Gledhill S., Hope R.G., Coy J.F., Girod A., McLauchlan J. (2003). Live Cell Analysis and Targeting of the Lipid Droplet-Binding Adipocyte Differentiation-Related Protein. J. Biol. Chem..

