# Supplementary material for: Four-and-a-Half LIM-Domain Protein 2 (FHL2) Induces Neuropeptide Y (NPY) in Macrophages in Visceral Adipose Tissue and Promotes Diet-Induced Obesity
Source: Int J Mol Sci. 2023 Oct 6;24(19):14943. doi: 10.3390/ijms241914943 (PMC10573629; doi:10.3390/ijms241914943)
Supplement: Supplementary file 1 [file ijms-24-14943-s001.zip › ijms-2653150-supplementary.pdf]

Supplementary Material

Four-and-a-Half LIM-Domain Protein 2 (FHL2) induces neuropeptide Y (NPY) in macrophages in visceral adipose tissue and promotes diet-induced obesity

Judith Sommer, Hanna Ehnis, Tatjana Seitz, Julia Schneider, Andreas B. Wild, Sandra Moceri, Christa Buechler, Aline Bozec, Georg F. Weber, Susanne Merkel, Ruth Beckervordersandforth, Alexander Steinkasserer, Roland Schüle, Jonel Trebicka, Arndt Hartmann, Anja Bosserhoff, Stephan von Hörsten, Peter Dietrich and Claus Hellerbrand

Supplementary Figure S1

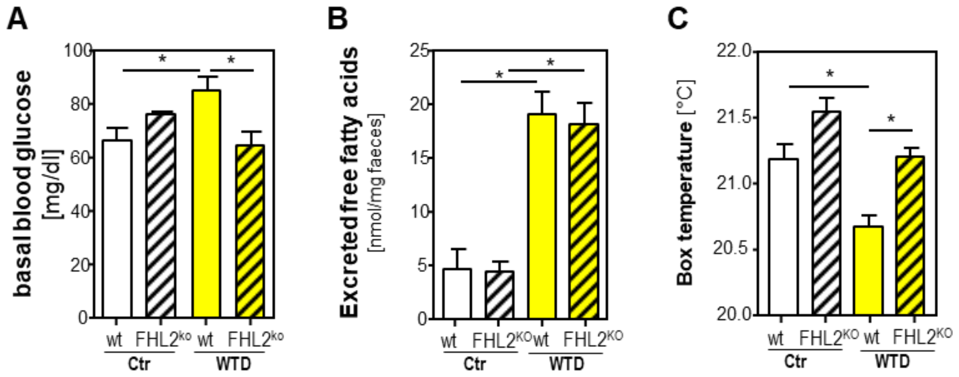

Figure S1 Wt and FHL2KO mice were fed with a Western-type diet (WTD) or standard chow (Ctr) for 18 weeks. Effect of FHL2 deficiency on (A) basal blood glucose concentration (B) excreted free fatty axids in animals feaces and (C) Box temperature in the cages (*: $p<0.05$).

Supplementary Figure S2

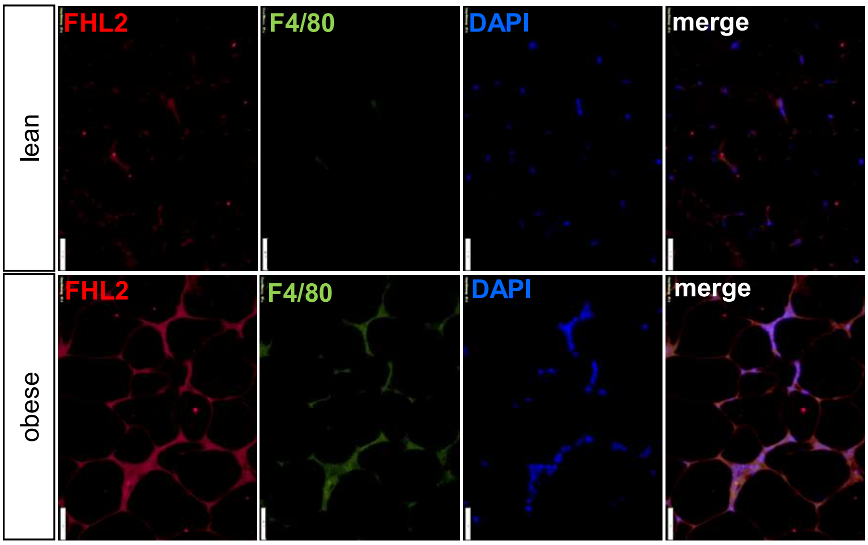

Figure S2 FHL2 expression in human visceral adipose tissue. Co-immunofluorescence staining of FHL2 (red) and F4/80 (green) in visceral adipose tissue of lean and obese humans (20X magnification).

Supplementary Figure S3

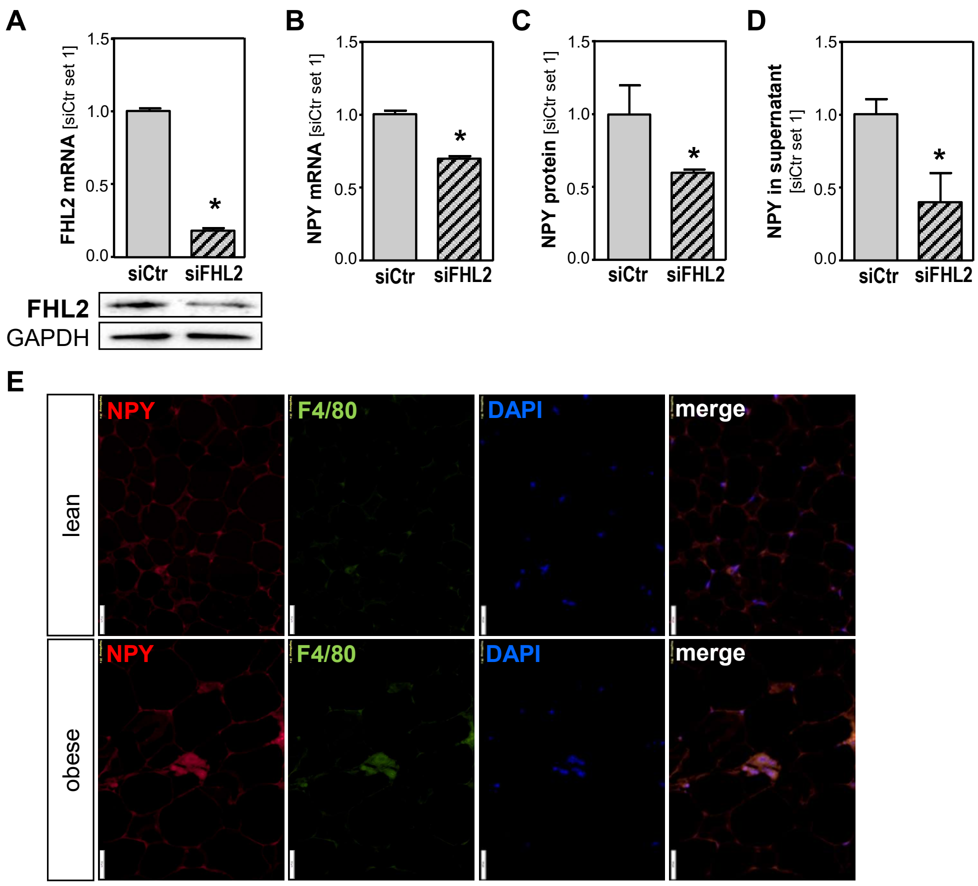

Figure S3 Effect of FHL2 depletion on NPY expression in macrophages and co-localization of FHL2 and NPY in human visceral adipose tissue. **(A)** FHL2 mRNA and protein expression, **(B)** NPY mRNA expression, **(C)** NPY protein level in cell lysates and **(D)** NPY levels in supernatant in primary murine peritoneal macrophages transfected with siPOOLs against FHL2 (siFHL2) or control pools (siCtr) for 72h (*: $p<0.05$). **(E)** Co-immunofluorescence staining of NPY (red) and F4/80 (green) in visceral adipose tissue of lean and obese humans (20X magnification).

Supplementary Figure S4

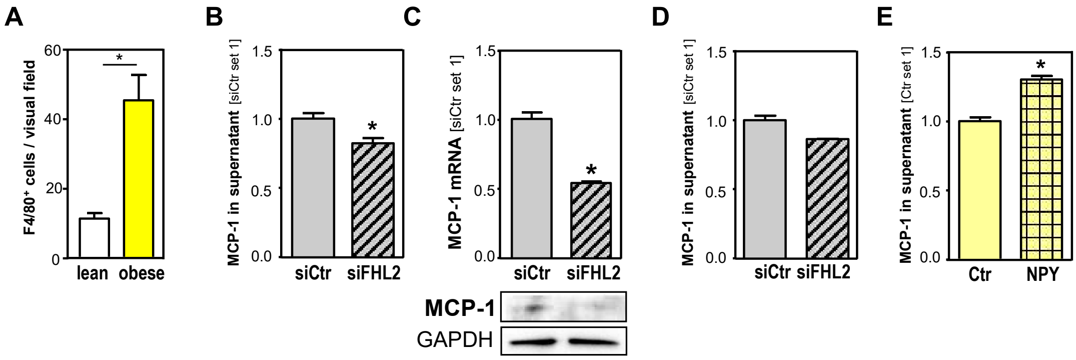

Figure S4 Comparison of macrophage infiltration in visceral adipose tissue of lean and obese humans and effect of FHL2 on MCP-1 expression in macrophages and adipocytes. **(A)** F4/80-positive cells in visceral adipose tissue of lean and obese humans. **(B)** MCP-1 level in the supernatant of Raw246.7 cells transfected with siPOOLs against FHL2 (siFHL2) or control pools (siCtr). **(C)** MCP-1 mRNA and protein levels and **(D)** MCP-1 level in the supernatant of primary murine peritoneal macrophages transfected with siPOOLs against FHL2 (siFHL2) or control pools (siCtr). **(E)** MCP-1 level in the supernatant of differentiated 3T3 cells treated with NPY (100 nM) for 24h (*: $p<0.05$).
